# Enhancing Nursing Students' Compassion, Empathy and Communication Through Mindfulness‐Based Meditation: A Mixed‐Methods Study

**DOI:** 10.1002/nop2.70325

**Published:** 2025-11-24

**Authors:** Tuba Sengul, Aleyna Özkan, Ayşe Eminoğlu, Noordeen Shoqirat, Charleen Singh, Deema Mahasneh, Holly Kirkland‐Kyhn

**Affiliations:** ^1^ School of Nursing, Koç University Istanbul Türkiye; ^2^ Department of Nursing, Faculty of Health Sciences Marmara University Istanbul Türkiye; ^3^ Faculty of Nursing, Faculty of Health Sciences, Higher Colleges of Technology Abudabi UAE; ^4^ Faculty of Nursing Mutah University Mutah Jordan; ^5^ California's College Town Davis California USA; ^6^ DNP‐FNP Program Sacramento California USA; ^7^ Nursing Department, College of Pharmacy and Health Sciences Ajman University Ajman UAE; ^8^ Betty Irene School of Nursing, UC Davis Sacramento California USA

**Keywords:** communication skills, compassion meditation, empathy, mental health well‐being, mindfulness‐based interventions, nursing students, patient‐centred care

## Abstract

**Aim:**

This study investigates the impact of mindfulness‐based compassion meditation on nursing students' compassion, emotional empathy and communication skills.

**Design:**

An explanatory sequential mixed‐methods design was employed. Quantitative data were collected through a quasi‐experimental approach, followed by qualitative data to gain deeper insights.

**Methods:**

The study was conducted during the 2023–2024 academic year with 53 nursing students from private and two public universities. Participants engaged in an 8‐week, 16‐session online mindfulness‐based compassion meditation programme structured around Kabat‐Zinn's Mindfulness‐Based Stress Reduction (MBSR) framework and Neff's Self‐Compassion Theory. Quantitative data were collected using the Compassion Competence Scale (CCS), Multidimensional Emotional Empathy Scale (MEES) and Communication Skills Scale (CSS) before and after the programme. Additionally, focus group discussions (FGDs) were conducted with 20 volunteers to explore their meditation experiences.

**Results:**

Students (*n* = 53) had a mean age of 21 years, with 92.5% being female, 73.6% having chosen the nursing profession voluntarily and 94.3% reporting that they had never received formal compassion training. Following the mindfulness‐based compassion meditation programme, a significant increase was observed in the scores of the Compassion Competence Scale (CCS), the Communication Skills Scale (CSS) and the subdimensions of communication, sensitivity and insight (*p* = 0.000–0.012). The Multidimensional Emotional Empathy Scale (MEES) scores also increased; however, this change was not statistically significant (*p* = 0.297). Qualitative findings revealed that participants expressed their meditation experiences under the themes of ‘Personal Reflections on Meditation’, ‘Self‐Compassion Awareness and Reflection’, ‘Emotional Awareness and Regulation’, ‘Empathic Approach in Relationships’, ‘Limitations and Challenges of Meditation’ and ‘Gains and Sustainability of Meditation’.

**Conclusion:**

Mindfulness‐based compassion meditation has been shown to enhance nursing students' compassion, empathy and communication skills, supporting their professional and emotional development. Integrating such interventions into structured nursing education programmes may increase individual awareness, prepare students for future work environments, strengthen professional resilience and promote patient‐centred care.

**Patient or Public Contribution:**

Mindfulness‐based compassion meditation has the potential to equip future nurses with essential skills that can improve their ability to manage stress, engage in compassionate care and enhance interdisciplinary collaboration. Expanding such programmes beyond nursing education and into clinical settings could further support healthcare professionals' well‐being and professional development, ultimately improving patient care quality.

## 1 Introduction

Mindfulness‐based interventions are mental training techniques that help individuals focus on the present moment non‐judgmentally and consciously (Zhang et al. 2021). The literature highlights the effectiveness of these interventions in reducing stress, anxiety and depression while promoting psychological well‐being (Burton et al. 2017; Goldberg et al. 2022). Additionally, mindfulness‐based practices have been shown to enhance essential skills such as self‐compassion, empathy and awareness among healthcare professionals, improve interdisciplinary communication and reduce burnout (Jiménez‐Picón et al. 2021; Green and Kinchen 2021; McConville et al. 2017).

Concepts such as ‘non‐judgement’, ‘patience’, ‘trust’ and ‘acceptance’, which are integral to mindfulness, are also fundamental qualities expected in nursing care (Walker and Mann 2016). Mindfulness‐based interventions have been found to enhance stress and emotion regulation skills, improve sensitivity towards patient care and support the psychological well‐being of nurses (Jiménez‐Picón et al. 2021; Green and Kinchen 2021). Compassion and self‐compassion are essential components of nursing care, directly influencing the quality of patient–nurse interactions (Akyol Bircan and Çakmak 2023; Uslu and Demir Korkmaz 2017). Research suggests that for nurses to provide compassionate care to their patients, they must first cultivate self‐compassion towards themselves (Raab 2014; Uslu and Demir Korkmaz 2017).

Empathy is a core element of patient‐centred care, facilitating trust‐based relationships between nurses and patients (Çıray Gündüzoğlu 2019; Çingöl et al. 2018). Studies indicate that nursing students generally exhibit high levels of compassion, with a significant correlation between their compassion levels and empathy tendencies (Özdelikara and Babur 2020). Nurses with higher self‐compassion levels demonstrate better empathy in patient care, allowing them to understand patients' emotional needs more effectively (Jiménez‐Picón et al. 2021; Guillaumie et al. 2017; Dilmaç Pınar and Ceylan 2024). In recent years, mindfulness‐based interventions have been widely integrated into nursing education. Research has shown that mindfulness techniques reduce stress, anxiety and depression levels while increasing awareness, self‐compassion and empathy among nursing students (Aloufi et al. 2021; van der Riet et al. 2018; Guillaumie et al. 2017; Song and Lindquist 2015). A meta‐analysis by Chen et al. (2021) also confirmed that mindfulness‐based interventions significantly reduce depression and anxiety levels while enhancing mindfulness among nursing students. Furthermore, studies suggest a positive relationship between self‐compassion and mindfulness levels in nursing students, with higher self‐compassion leading to improved empathy, communication skills and compassionate patient care (Yaman et al. 2022; Dilmaç Pınar and Ceylan 2024).

In Turkey, where nursing education increasingly incorporates evidence‐based psychosocial interventions, formal integration of mindfulness and compassion‐based training into undergraduate curricula remains limited (Yüksel and Bahadır Yılmaz 2020; Erkin and Şenuzun Aykar 2021; Taş Arslan et al. 2023). Addressing this gap may enhance nursing students' preparedness for patient‐centred care and improve their professional resilience (Yüksel and Bahadır Yılmaz 2020; Liu et al. 2024).

The literature includes numerous systematic reviews and meta‐analyses demonstrating the effectiveness of mindfulness‐based interventions in improving awareness, self‐efficacy and empathy in nursing students (McConville et al. 2017; Sumantry and Stewart 2021). However, no study has simultaneously examined the effects of mindfulness‐based compassion meditation on compassion, emotional empathy and communication skills in nursing students. Unlike general mindfulness practices, compassion meditation focuses on cultivating kindness, non‐judgement and emotional connection, making it particularly relevant for nursing education. Given that nursing requires both cognitive empathy (understanding patients' emotions) and affective empathy (sharing their feelings), compassion meditation is expected to strengthen both self‐compassion and interpersonal compassion, leading to better patient‐centred care (Jiménez‐Picón et al. 2021; Green and Kinchen 2021; Raab 2014). Thus, this study aims to investigate how mindfulness‐based compassion meditation enhances nursing students' compassion, emotional empathy and communication skills, providing insights into its potential integration into nursing education and clinical practice.

## 2 Material and Method

### 2.1 Aim

This study aims to explore the experiences of nursing undergraduate students participating in mindfulness‐based compassion meditation and to examine its role in enhancing their compassion, emotional empathy and communication levels.

### 2.2 Design of the Study

This study employed a mixed‐methods approach, integrating quantitative and qualitative data collection and analysis. The research followed an explanatory sequential mixed‐methods design, in which quantitative data were collected first, followed by qualitative data collection and interpretation to provide deeper insights into the findings. The quantitative phase utilised a quasi‐experimental design with pre‐test and post‐test measurements to assess student compassion, emotional empathy and communication skills changes. The qualitative phase was guided by two primary theoretical frameworks explaining mindfulness‐based meditation's individual and interpersonal effects. The mindfulness component of the study was structured within Kabat‐Zinn's (1990) Mindfulness‐Based Stress Reduction (MBSR) Program, which describes the effects of mindfulness practice on cognitive regulation, stress management and emotional awareness. The compassion meditation component was based on Neff's Self‐Compassion Theory (2023), which explains how individuals cultivate self‐compassion and how it enhances compassionate communication in interpersonal relationships. By integrating these two models, this study aimed to explore the mindfulness‐based compassion meditation intervention's potential effects on nursing students' self‐awareness, compassion and communication skills.

### 2.3 Participants and Setting

The study sample consisted of nursing students in their 1st, 2nd, 3rd and 4th years from one private and two public universities in Turkey during the 2023–2024 academic year. The sample size was determined using G*Power 3.1.9.4 for a multiple linear regression model, assuming a moderate effect size (*f* ^2^ = 0.15), *α* = 0.05, 80% power and six predictors. The six predictors were: (Akyol Bircan and Çakmak 2023) age, (Aloufi et al. 2021) gender, (Bidik and Sisman 2024) year of study, (Braun and Clarke 2006) voluntary choice of the nursing profession, (Burton et al. 2017) prior formal training in compassion and (Caruso and Mayer 1998) baseline scores on the outcome variable. These predictors were selected based on evidence that mindfulness‐based interventions improve emotional intelligence, self‐compassion and professional caring behaviours among nursing students and healthcare professionals (Jiménez‐Picón et al. 2021; Bidik and Sisman 2024; Vardar İnkaya et al. 2025). This calculation indicated a required sample size of 55 students to ensure adequate statistical power and reproducibility.

#### 2.3.1 Inclusion Criteria

- Enrollment in a nursing undergraduate programme
- No prior participation in a mindfulness‐based compassion meditation programme
- Voluntary participation and provision of written informed consent

#### 2.3.2 Exclusion Criteria

- Missing one or more meditation sessions (non‐attendance)
- Withdrawing from the study before completion

A total of 55 students who met the criteria voluntarily participated in the study. The mindfulness‐based compassion meditation programme lasted 8 weeks, with two sessions per week, totalling 16 sessions. Two students were excluded during the intervention due to missing one or more sessions. As a result, the quantitative phase of the study was completed with 53 students. For the qualitative phase, 20 students were selected from the 53 participants based on their willingness to participate and three separate focus group discussions (FGDs) were conducted.

### 2.4 Data Collection

#### 2.4.1 Student Information Form

A 14‐item questionnaire developed by the researchers was used to collect demographic and general information about participants, including age, gender and academic year.

#### 2.4.2 Compassion Competence Scale (CCS)

Developed by Lee and Seomun (2016) and adapted into Turkish by Çiftçi and Aras (2021), this scale consists of three subdimensions: ‘Communication’, ‘Sensitivity’ and ‘Insight’. It is a 5‐point Likert‐type scale, with each item rated from 1 (strongly disagree) to 5 (strongly agree). A higher total score indicates a higher level of compassion competence. The Cronbach's *α* coefficient for the Turkish version was reported as 0.795, while it was calculated as 0.71 in this study.

#### 2.4.3 Multidimensional Emotional Empathy Scale (MEES)

Developed by Caruso and Mayer (1998) and adapted into Turkish by Turan et al. (2021), this scale evaluates individuals' empathic abilities in multiple dimensions. It consists of 30 items, with total scores ranging from 30 to 150, where higher scores indicate higher levels of empathy. The original Cronbach's *α* coefficient was reported as 0.88, while it was calculated as 0.75 in this study.

#### 2.4.4 The Communication Skills Scale (CSS)

Developed by Fidan Korkut (1996), this scale assesses individuals' communication skills. It is a 5‐point Likert‐type scale (5 = always, 1 = never) comprising 25 items. The total score ranges from 25 to 125, with higher scores indicating better communication skills. The original Cronbach's *α* coefficient was reported as 0.80, while it was calculated as 0.73 in this study.

### 2.5 Qualitative Measurements

Following the quantitative data analysis, FGDs were conducted using a semi‐structured question guide to gain deeper insight into participants' experiences with mindfulness‐based compassion meditation. The FGDs were structured based on the core principles of Kabat‐Zinn's Mindfulness‐Based Stress Reduction (MBSR) model, emphasising breath awareness, body scanning, non‐judgemental awareness and present‐moment attention. Additionally, Neff's Self‐Compassion Theory (2023) was integrated into the qualitative framework to explore how participants developed self‐compassion and its influence on their interpersonal relationships and communication skills. Each FGD session consisted of 6–8 participants, and all discussions were audio‐recorded and transcribed verbatim for analysis. The average duration of each session was approximately 45 min. During FGDs, participants were asked the following questions:

- How did you experience the meditation process overall?
- How did mindfulness‐based compassion meditation affect how you express compassion towards yourself or others?
- How did the meditation process influence your ability to empathise? Did you feel increased empathy towards your patients or those around you?
- Did the meditation practices bring any changes to your communication skills?
- When faced with challenging emotions, did meditation techniques help you manage them?
- What was your experience of staying present and observing your thoughts without judgement?
- What aspects of this meditation programme were most beneficial or challenging?
- Do you plan to integrate mindfulness‐based meditation practices into your personal or professional life in the future?

### 2.6 Implementation Phase

The study was conducted via Zoom, an online platform. Students who agreed to participate received detailed information via email about the purpose of mindfulness‐based compassion meditation, the overall process and online participation guidelines. Although the intervention was conducted online, participant engagement was actively monitored, and attendance rates were recorded. At the beginning of the programme, participants attended a 2‐h theoretical training session introducing the fundamental principles of mindfulness‐based compassion meditation. This session covered meditation's physiological and neurological effects, recommended sitting postures, strategies for managing distractions and general session guidelines. As mentioned in the literature, a structured 8‐week programme consisting of 16 sessions was implemented following this introductory session (Kabat‐Zinn 1990). Each session lasted approximately 1 h, allowing sufficient time for meditation practice and participant feedback. The programme was conducted twice weekly, with fixed sessions on Tuesdays and Fridays from 9:00 PM to 10:00 PM, scheduled based on student preferences determined through a preliminary survey. During the sessions, mindfulness‐based meditation techniques such as breath awareness, body scanning, non‐judgemental awareness and present‐moment attention were practiced. The self‐compassion meditation component guided participants in developing compassion towards themselves and others, internalising the sense of compassion and observing their thoughts without judgement. The meditation practice lasted approximately 30–35 min, followed by a 15–20 min discussion period where participants could share their experiences and provide feedback. At the end of each session, participants were allowed to share their experiences and provide feedback. The meditation sessions were led voluntarily by an ICF‐Accredited Coaching Education Program‐certified instructor, and participant attendance was regularly monitored. Throughout the programme, email reminders were sent to participants before each session. Upon completion of the programme, a post‐test was administered to collect quantitative data. Additionally, FGDs were conducted using a structured question guide to gain deeper insights into participants' meditation experiences.

Participants were encouraged to practice individual meditation for at least 15 min daily throughout the programme; however, this was not assigned as a mandatory task. No additional materials or compulsory exercises were required, but participants were recommended to read books on meditation and self‐compassion. Before and after each session, participants were invited to share brief feedback on their experiences, their emotions during meditation and any changes in their awareness. This 15–20 min sharing process at the end of each session allowed participants to express their reflections and learn from each other in a group setting. Participants could also anonymously submit written experience reports and feedback for further reflection. To facilitate the integration of mindfulness practices into patient care, participants were encouraged to apply mindfulness techniques in clinical settings. For instance, they were advised to practice short‐breath awareness exercises before communicating with patients. However, the extent to which participants incorporated these practices into their routines was left to their discretion. Throughout the programme, feedback was collected to assess how meditation influenced personal awareness and professional applications.

**FIGURE 1:**
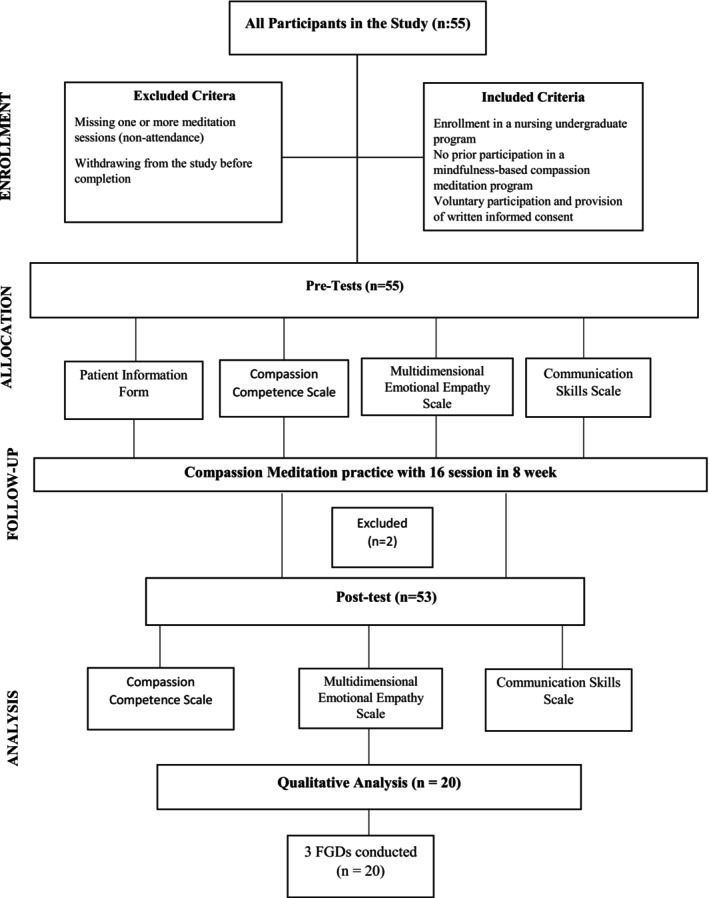
Consort diagram.

The flowchart of the study is presented in Figure 1.

### 2.7 Data Analysis

#### 2.7.1 Quantitative Data Analysis

Statistical analyses of quantitative data were conducted using IBM SPSS Statistics 24. Descriptive statistics, including mean, standard deviation, minimum and maximum values, were used for continuous variables, while frequency and percentage distributions were used for categorical variables. To assess the differences between pre‐test and post‐test scores, the Paired Samples *t*‐test was performed. The dependent variables included the Compassion Competence Scale (CCS), the Multidimensional Emotional Empathy Scale (MEES) and the Communication Skills Scale (CSS). Since this study employed a quasi‐experimental pre–post design without randomisation, and only participants who completed the entire intervention and both assessments were included in the analysis, an intention‐to‐treat (ITT) analysis was not applicable. This approach is consistent with methodological recommendations for quasi‐experimental designs where dropouts are excluded from the final analysis (Dettori and Norvell 2020). The quantitative findings were reported following the Strengthening the Reporting of Observational Studies in Epidemiology (STROBE) guidelines (von Elm et al. 2007).

#### 2.7.2 Qualitative Data Analysis

Qualitative data from FGDs were analysed using Braun and Clarke's (2006) six‐phase thematic analysis method. This process involved familiarisation with the data, where researchers immersed themselves in the transcripts to gain a comprehensive understanding. Next, initial codes were generated to identify meaningful patterns systematically. These codes were then organised to search for themes, grouping similar patterns. Researchers refined the themes in the reviewing themes phase and ensured their coherence. This was followed by defining and naming themes, where each theme was assigned clear labels and descriptions to encapsulate its meaning. Finally, in the reporting findings phase, the results were compiled into a structured narrative supported by participant quotations to enhance credibility and depth. An inductive approach was adopted for theme identification, allowing themes to emerge directly from the data without predefined categories. Two independent researchers conducted the initial coding to ensure rigour, and any discrepancies were resolved through consensus discussions. The qualitative findings were reported following the Consolidated Criteria for Reporting Qualitative Research (COREQ) checklist (Tong et al. 2007), ensuring transparency and methodological integrity.

### 2.8 Trustworthiness

Lincoln and Guba's (1985) four fundamental criteria—credibility, transferability, dependability and confirmability—were applied to ensure the trustworthiness of the qualitative phase. Credibility was established through audio‐recorded interviews and timely transcriptions, ensuring the reliability of the data. Researcher triangulation was employed, where multiple researchers conducted independent coding, followed by consensus discussions, enhancing the validity of the findings. Additionally, direct participant quotations were incorporated to reinforce thematic interpretations. Transferability was ensured by providing detailed descriptions of the research process, participant characteristics and study setting, enabling other researchers to assess the applicability of the findings within their contexts. Dependability was addressed by maintaining a transparent data analysis process and systematically documenting all methodological decisions and analytical steps. Finally, confirmability was achieved through continuous reflexivity, minimising researcher bias during data interpretation. A systematic peer review process was also implemented to maintain objectivity and ensure that the findings accurately reflected participants' experiences.

### 2.9 Ethical Considerations

Ethical approval for this study was obtained from the Private Foundation University Ethics Committees (Approval No: 2022.337.IRB3.153). In addition to institutional approvals, informed consent was obtained from all participants before the study commenced. Participants were provided detailed information regarding the study's purpose, procedures, potential risks and benefits. They were assured that participation was entirely voluntary, that they could withdraw from the study without consequences, and that confidentiality and anonymity would be strictly maintained throughout the research process. All personal data were securely stored, with access restricted to the research team. All identifying information was removed during the final data analysis and reporting to ensure participant privacy. Furthermore, the study adhered to the ethical principles outlined in the Declaration of Helsinki, upholding the highest research integrity and participant protection standards.

## 3 Results

### 3.1 Quantitative Findings

The demographic characteristics of the participants (Table 1) indicate that the majority of the nursing students were female (92.5%) with a mean age of 21.20 ± 1.39 years (range: 19–26). Most participants were in their second year of nursing education (62.3%), while the remaining students were distributed across the first (7.5%), third (22.6%) and fourth (7.5%) years. Regarding self‐perceptions, 92.5% of the students considered themselves compassionate, while only 5.7% had previously received any formal compassion training. The students' self‐perceived scores before meditation revealed that their overall compassion level (6.90 ± 1.40), self‐compassion level (5.39 ± 1.83) and overall communication skills (6.58 ± 1.68) were at moderate levels. However, emotional empathy (7.13 ± 1.67) and empathy towards others/patients (7.22 ± 1.56) were above average, suggesting that students already had a heightened awareness of empathy before the intervention (Table 1).

**TABLE 1:** Demographic and baseline characteristics of nursing students (*n* = 53).

Characteristics	*n*	(%)
Gender		
Female	49	92.5
Male	4	7.5
Class		
1st year	4	7.5
2nd year	33	62.3
3rd year	12	22.6
4th year	4	7.5
Self‐perceived compassion	49	92.5
Formal compassion training (yes)	3	5.7

	*X* ± SD	Min–max
Age (years)	21.20 ± 1.39	19–26
Overall Compassion Level^a^	6.90 ± 1.40	1–10
Self‐Compassion Level^a^	5.39 ± 1.83	1–10
Emotional Empathy Level^a^	7.13 ± 1.67	2–10
Empathy toward Others/Patients^a^	7.22 ± 1.56	2–10
Overall Communication Skills^a^	6.58 ± 1.68	1–10
Communication with Others/Patients^a^	6.67 ± 1.64	1–10

^a^ All self‐perceived scores are on a 1–10 scale.

Additional descriptive statistics for MEES post‐test scores indicated a skewness of −1.70 and a kurtosis of 4.05, confirming a ceiling effect where the majority of participants scored near the upper limit of the scale.

**FIGURE 2:**
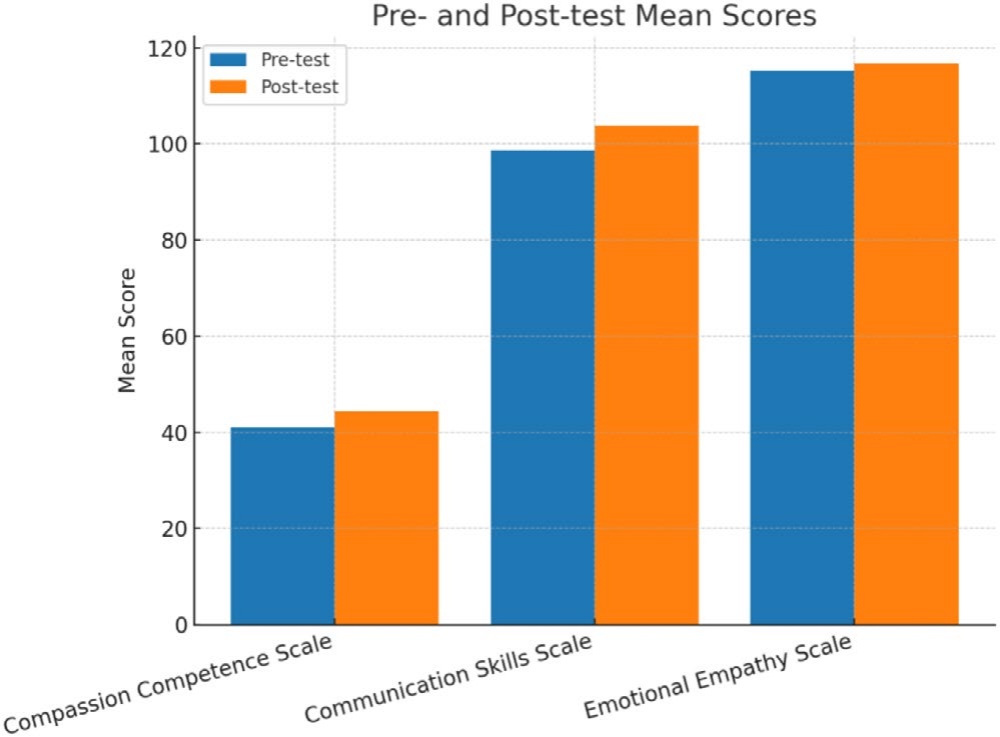
Pre‐ and post‐test mean scores for the Compassion Competence Scale (CCS), Communication Skills Scale (CSS) and Multidimensional Emotional Empathy Scale (MEES).

Table 2 presents the pre‐ and post‐test mean scores for the Compassion Competence Scale (CCS), the Communication Skills Scale (CSS) and the Multidimensional Emotional Empathy Scale (MEES) (Figure 2). The results demonstrated a statistically significant increase in compassion competence after the intervention (pre‐test: 41.07 ± 5.20; post‐test: 44.37 ± 4.19; mean difference = 3.30, 95% CI [1.99, 4.61]; *t* = −5.065, *p* < 0.001). Similarly, communication skills significantly improved (pre‐test: 98.73 ± 11.35; post‐test: 103.81 ± 9.37; mean difference = 5.08, 95% CI [2.31, 7.85]; *t* = −3.680, *p* = 0.001). The subdimensions of the CCS also showed significant improvement. The communication subscale increased from 18.22 ± 2.67 to 19.50 ± 2.35 (mean difference = 1.28, 95% CI [0.57, 1.99]; *t* = −3.638, *p* = 0.001), the sensitivity subscale from 12.13 ± 1.74 to 12.83 ± 1.60 (mean difference = 0.70, 95% CI [0.15, 1.25]; *t* = −2.610, *p* = 0.012) and the insight subscale from 10.71 ± 1.89 to 12.03 ± 1.45 (mean difference = 1.32, 95% CI [0.80, 1.84]; *t* = −4.984, *p* < 0.001). In contrast, the overall emotional empathy score did not change statistically (pre‐test: 115.28 ± 11.87; post‐test: 116.71 ± 11.58; mean difference = 1.43, 95% CI [−1.29, 4.15]; *t* = −1.054, *p* = 0.297) (Table 2).

**TABLE 2:** Pre‐ and post‐test scores for CCS, CSS and MEES.

Scale/Subscale	Pre‐test Mean ± SD	Post‐test Mean ± SD	Mean difference	95% CI for difference	*t*	*p*
CCS total	41.07 ± 5.20	44.37 ± 4.19	3.30	[1.99, 4.61]	−5.065	< 0.001
Communication	18.22 ± 2.67	19.50 ± 2.35	1.28	[0.57, 1.99]	−3.638	0.001
Sensitivity	12.13 ± 1.74	12.83 ± 1.60	0.70	[0.15, 1.25]	−2.610	0.012
Insight	10.71 ± 1.89	12.03 ± 1.45	1.32	[0.80, 1.84]	−4.984	< 0.001
CSS total	98.73 ± 11.35	103.81 ± 9.37	5.08	[2.31, 7.85]	−3.680	0.001
MEES total	115.28 ± 11.87	116.71 ± 11.58	1.43	[−1.29, 4.15]	−1.054	0.297

Abbreviations: CCS, Compassion Competence Scale; CSS, Communication Skills Scale; MEES, Multidimensional Emotional Empathy Scale.

### 3.2 Qualitative Findings

**FIGURE 3:**
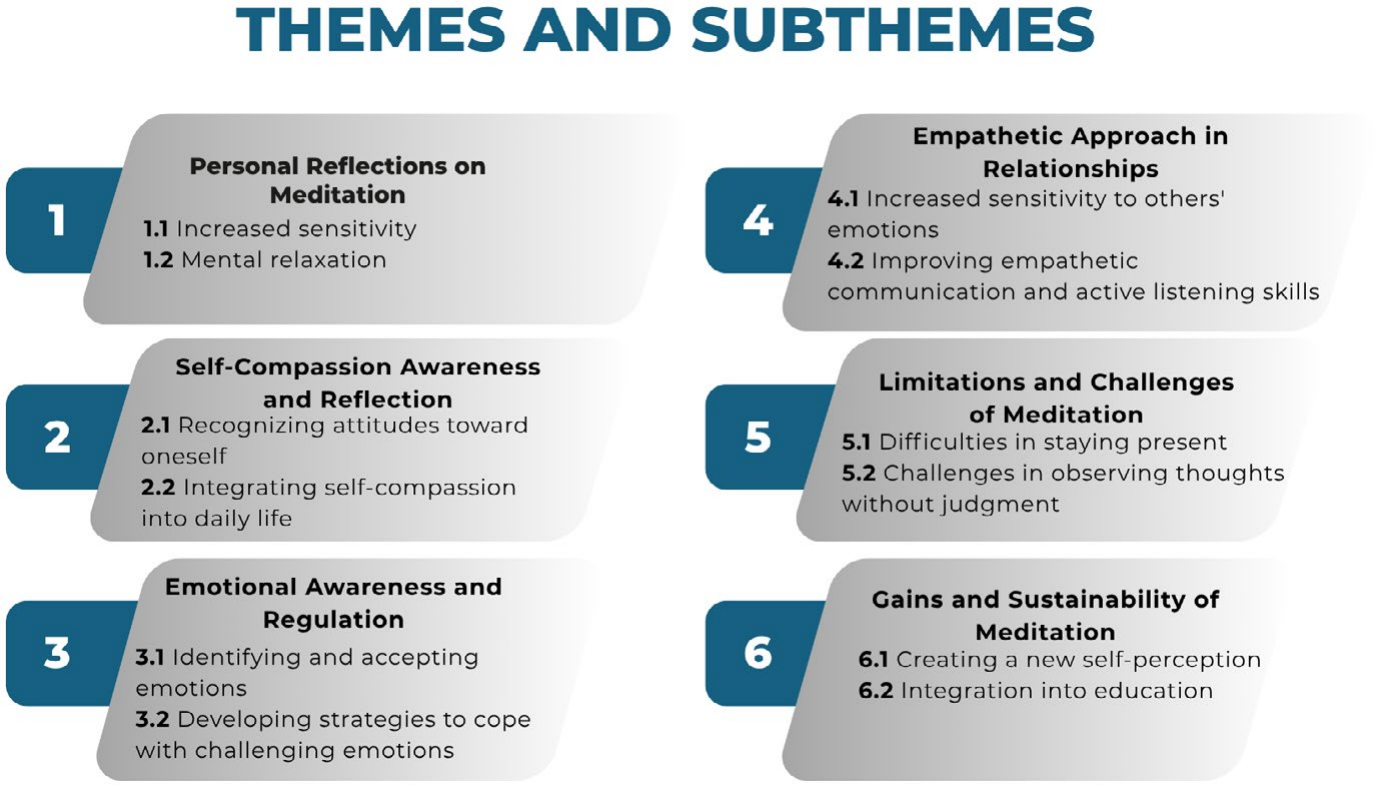
Themes and subthemes identified in the study.

The qualitative analysis identified six main themes reflecting participants' experiences with mindfulness‐based compassion meditation (see Figure 3). The first theme, ‘Personal Reflections on Meditation’, includes increased sensitivity and mental relaxation subthemes, describing participants' heightened awareness and calm experiences. The second theme, ‘Self‐Compassion Awareness and Reflection’, consists of recognising attitudes towards oneself and integrating self‐compassion into daily life, highlighting participants' reflections on self‐perception and behaviours. The third theme, ‘Emotional Awareness and Regulation’, involves identifying and accepting emotions and developing strategies to cope with challenging emotions, demonstrating how participants engaged with their emotional experiences. The fourth theme, ‘Empathetic Approach in Relationships’, encompasses increased sensitivity to others' emotions and improving empathetic communication and active listening skills, describing changes in participants' interpersonal interactions. The fifth theme, ‘Limitations and Challenges of Meditation’, includes difficulties in staying present and challenges in observing thoughts without judgement, reflecting obstacles participants faced during meditation practice. The final theme, ‘Gains and Sustainability of Meditation’, consists of creating a new self‐perception and integration into education, capturing participants' perspectives on the continuity and applicability of meditation practices (Figure 3).

The qualitative analysis identified six main themes reflecting participants' experiences with mindfulness‐based compassion meditation (see Figure 3). In Figure 3, each main theme is presented in a separate coloured box, with its related subthemes listed underneath. Arrows between some themes indicate conceptual overlaps, showing how participants' experiences are interconnected across different thematic areas. This visual structure highlights the relationships between themes, such as the link between emotional awareness and empathic approaches to relationships, or between self‐compassion and sustainable meditation practices.

### 3.3 Theme 1: Personal Reflections on Meditation

Participants expressed how meditation influenced their self‐awareness, particularly under the ‘Increased Sensitivity’ subthemes and ‘Mental Relaxation’.

#### 3.3.1 Subtheme: 1.1 Increased Sensitivity

When I practice meditation, I become more aware of the people around me throughout the day. (F, A21, P3)

#### 3.3.2 Subtheme: 1.2 Mental Relaxation

I had never tried it before. I wondered why I hadn't. I actually started to feel peaceful. (F, A22, P8)

During meditation, I feel calmer, and I think I am mentally more relaxed. (M, A23, P11)

### 3.4 Theme 2: Self‐Compassion Awareness and Reflection

Participants shared how meditation enhanced their self‐compassion and compassion towards others.

#### 3.4.1 Subtheme: 2.1 Recognising Attitudes Towards Oneself

I realized that I am compassionate toward others, but not toward myself. I noticed that I criticize myself harshly. (F, A21, P13)

Before this experience, I never noticed how often I blamed myself for things beyond my control. Meditation taught me to approach my struggles with more patience and less self‐judgment. (F, A22, P5)

#### 3.4.2 Subtheme: 2.2 Integrating Self‐Compassion Into Daily Life

After meditation, I feel more compassionate toward people, my patients, and even all living beings. (F, A23, P10)

I used to get frustrated when patients were uncooperative, but now I try to understand their emotions rather than reacting immediately. I realize that they are going through their own struggles, and I need to be patient. (F, A24, P7)

I have started to truly listen when people speak, instead of preparing my response in my mind. I think this has made my conversations more meaningful. (M, A23, P11)

### 3.5 Theme 3: Emotional Awareness and Regulation

Participants highlighted how meditation improved their ability to recognise and accept difficult emotions, helping them regulate their emotional responses more effectively.

#### 3.5.1 Subtheme: 3.1 Identifying and Accepting Emotions

When I practice compassion meditation, I feel calmer and can manage my emotions better. (M, A21, P16)

I think meditation helps reduce stress and anxiety during difficult times. (F, A21, P6)

I think meditation helps reduce stress and anxiety during difficult times. I used to feel paralyzed by negative thoughts, but now I remind myself that emotions come and go, and I do not have to get stuck in them. (F, A22, P20)

#### 3.5.2 Subtheme: 3.2 Developing Strategies to Cope With Challenging Emotions

Facing one's own inner emotions is very difficult, but meditation makes it easier. It feels like talking to myself. (M, A24, P18)

### 3.6 Theme 4: Empathetic Approach in Relationships

This theme reflected the impact of meditation on participants' social interactions.

#### 3.6.1 Subtheme: 4.1 Increased Sensitivity to Others' Emotions

After meditation, I realized that I approach people and my patients with more empathy. (M, A21, P16)

Before, I used to focus mostly on completing tasks during patient care. Now, I try to pay attention to their emotions too. I ask myself, ‘How would I feel in their place?’ and this helps me provide more compassionate care. (F, A23, P14)

#### 3.6.2 Subtheme: 4.2 Improving Empathetic Communication and Active Listening Skills

I think I can remain calmer in conversations and listen better. (F, A23, P15)

### 3.7 Theme 5: Limitations and Challenges of Meditation

Participants expressed difficulties in maintaining mindfulness without judgement.

#### 3.7.1 Subtheme: 5.1 Difficulties in Staying Present

As a student, it's so hard to focus on just one thing. My mind constantly drifts to something else, and I catch myself thinking about different things. (F, A22, P2)

Observing my thoughts is difficult. My mind keeps jumping to the past or future. (F, A21, P17)

I try to concentrate, but before I know it, I'm thinking about assignments, exams, or something that happened earlier in the day. It's difficult to quiet my mind. (F, A23, P10)

At first, I felt like I was failing at meditation because I couldn't stop my mind from drifting. But I've realized that bringing my attention back, even after getting distracted, is also part of the process. (M, A22, P12)

#### 3.7.2 Subtheme: 5.2 Challenges in Observing Thoughts Without Judgement

It is very difficult to watch my thoughts without judging or blaming myself. During meditation, I immediately start evaluating whatever comes to my mind, and I struggle to stop this. (F, A22, P19)

### 3.8 Theme 6: Gains and Sustainability of Meditation

Participants reflected on the long‐term benefits of meditation and its potential for continued practice.

#### 3.8.1 Subtheme: 6.1 Creating a New Self‐Perception

Meditation has been very beneficial for me. It helped me cope with difficulties and create a new self. (F, A22, P4)

Before meditation, I often felt trapped by my negative thoughts. Now, I realize that I do not have to believe every thought that comes into my mind. I have learned to observe them and let them pass rather than letting them control me. (F, A21, P3)

#### 3.8.2 Subtheme: 6.2 Integration Into Education

I think practicing meditation with professional guidance is more effective than doing it alone. (F, A22, P4)

I really wish these kinds of practices were included in classes, or even offered as an elective course in our education. (F, A23, P14)

Whenever I tried meditating on my own before this program, I struggled to stay focused. Having a structured program and professional guidance made a huge difference. I actually learned how to meditate properly, rather than just sitting in silence. (F, A22, P1)

## 4 Discussion

This study examined the effects of mindfulness‐based compassion meditation on nursing students' compassion, emotional empathy and communication levels. Quantitative findings revealed a significant increase in the post‐meditation Compassion Competence Scale (CCS) and the Communication Skills Scale (CSS) scores. In contrast, no statistically significant difference was found in Multidimensional Emotional Empathy Scale (MEES) scores. Additionally, there was a considerable improvement in the subdimensions of compassion, including communication, sensitivity and insight. These results suggest that meditation contributed to developing participants' self‐compassion and interpersonal compassion, ultimately enhancing their communication skills. Qualitative findings supported the quantitative results by demonstrating that meditation increased self‐awareness, facilitated emotional regulation and improved empathic communication among participants.

When evaluating students' initial self‐perceptions, their overall compassion level (6.90 ± 1.40), self‐compassion level (5.39 ± 1.83) and communication skills (6.58 ± 1.68) were found to be at moderate levels, while their emotional empathy (7.13 ± 1.67) and empathy towards others/patients (7.22 ± 1.56) were above average. These findings indicate that, at baseline, students already had a high awareness of empathy, whereas self‐compassion and communication skills appeared to be areas needing further development. This is a significant observation, as previous research frequently associates nursing students with strong empathic abilities due to the patient‐centred nature of the profession (Jiménez‐Picón et al. 2021; Guillaumie et al. 2017). However, the relatively lower self‐compassion levels suggest that meditation played a key role in increasing students' awareness and improvement in this domain, reinforcing the literature's emphasis on self‐compassion as a prerequisite for compassionate patient care (Raab 2014; Uslu and Demir Korkmaz 2017).

The observed increase in compassion and communication levels in quantitative results was also reflected in qualitative data, particularly in the Self‐Compassion Awareness and Reflection and Empathic Approach in Relationships. Participants expressed that meditation helped them develop a more understanding and compassionate attitude towards themselves, positively influencing their everyday interactions. They also reported an improved ability to communicate empathetically with patients, suggesting increased self‐compassion extended to their interpersonal relationships. Additionally, students highlighted that their enhanced awareness helped them manage clinical–patient interactions more effectively, which aligns with prior studies demonstrating that mindfulness‐based interventions improve compassion and communication skills among healthcare professionals (Jiménez‐Picón et al. 2021; Green and Kinchen 2021; Guillaumie et al. 2017).

The lack of a significant change in emotional empathy scores contrasts with some studies in the literature (McConville et al. 2017). One possible explanation is that students already had high levels of emotional empathy at baseline, creating a ceiling effect that limited the potential for further improvement. Given that emotional empathy (7.13 ± 1.67) was among the highest pre‐test scores, the lack of significant post‐meditation change in this domain may reflect an already strong foundation rather than a failure of the intervention. Additionally, some studies suggest that Generation Z exhibits higher empathy levels than previous generations (Karakuttikaran and Kolachina 2024). Nevertheless, the literature indicates that empathic skills may require more prolonged and intensive interventions to develop (Raab 2014; McConville et al. 2017).

A key theme from the qualitative findings was Personal Reflections on Meditation, where participants described experiencing mental relaxation and increased sensitivity. This aligns with previous research demonstrating that mindfulness‐based interventions effectively reduce stress and anxiety (Chen et al. 2019; van der Riet et al. 2018). Additionally, students encountered challenges in maintaining mindfulness, as highlighted in the theme Limitations and Challenges of Meditation. They reported difficulties staying present in the moment and observing their thoughts non‐judgmentally, consistent with studies showing that mindfulness practices require consistent effort and patience to be effective (Kabat‐Zinn 2016). Academic workload, exams and time constraints were frequently cited as barriers to fully engaging with meditation, emphasising the need for structured and sustainable mindfulness programmes in nursing education.

Although this study did not directly measure the impact of meditation on patient care, it is well‐established in the literature that compassion, empathy and communication skills significantly influence the quality of patient care (Özdelikara and Babur 2020). Participants' qualitative reports of enhanced empathic communication and self‐awareness suggest that these improvements may indirectly contribute to better clinical interactions. Active listening, sensitivity and compassionate engagement with patients are fundamental aspects of effective nurse–patient communication and mindfulness‐based compassion meditation has the potential to support these critical skills in clinical practice. Finally, students acknowledged the benefits of the meditation programme but stressed the importance of structured, professionally guided and continuous training to ensure its sustainability. Some participants suggested integrating mindfulness‐based interventions into elective courses or academic curricula would make such practices more systematic and practical. This finding aligns with previous research emphasising the importance of incorporating mindfulness into healthcare education (Walker and Mann 2016; Green and Kinchen 2021). Future research should explore the long‐term impact of mindfulness‐based compassion meditation on both nursing students and practicing nurses, particularly in clinical settings, to assess its sustained effects on professional competencies and patient care outcomes.

## 5 Limitations

This study has several limitations. First, the sample consisted of a limited number of nursing students, which restricts the generalizability of the findings to students. Future studies should include a larger sample to enhance the robustness of the results. Second, the study duration was limited to 8 weeks, preventing an assessment of the long‐term effects of meditation. Longitudinal follow‐up studies are needed to comprehensively examine the impact of mindfulness‐based meditation on professional skills over time. Third, the online nature of this study did not allow for a comparative evaluation with in‐person mindfulness programmes. Future research should explore whether mindfulness programmes in clinical settings affect stress management and patient interactions differently than online interventions. Additionally, this study was conducted without a control group, which limits causal inferences. While the findings indicate positive effects of mindfulness‐based compassion meditation, the absence of a control group makes it difficult to determine whether improvements resulted solely from the intervention or other factors. Future studies should incorporate randomised controlled trials (RCTs) to enhance methodological rigour. Furthermore, the online implementation may have influenced participant engagement and meditation depth. Face‐to‐face mindfulness sessions facilitate stronger peer interactions and guided reflections, potentially amplifying the benefits. Future research should compare online and in‐person mindfulness‐based interventions to assess differences in effectiveness and engagement. In addition, the internal consistency coefficients obtained in this study were slightly lower than those reported in the original validation studies. These values were close to the acceptable threshold for research purposes and should be interpreted with caution. Future studies with larger and more diverse samples are recommended to confirm the reliability findings. Finally, the lack of a significant change in emotional empathy levels suggests that enhancing empathic skills may require more extensive and long‐term interventions. Future studies should evaluate mindfulness‐based interventions over an extended period to determine their potential for fostering an empathic approach in patient care within nursing education.

## 6 Conclusions

Mindfulness‐based compassion meditation can help nursing students enhance their empathy, non‐judgemental awareness and communication skills, supporting a more compassionate approach to patient care. Specifically, this practice fosters greater self‐compassion and compassion towards others while facilitating empathetic communication and active, non‐judgemental listening. However, mindfulness practices may require regular implementation and professional guidance to be effective. Therefore, integrating mindfulness‐based interventions into nursing education and supporting them with structured programmes is essential. Incorporating such practices into the curriculum may help students better manage their emotional challenges in clinical settings. The skills acquired through this training can play a crucial role during students' academic years and in improving patient care quality throughout their professional careers. Additionally, to extend these benefits to healthcare professionals, it is recommended that hospitals implement regular mindfulness programmes. These initiatives could help healthcare professionals develop a more conscious, compassionate and empathetic approach to patient interactions while strengthening interdisciplinary communication.

## Author Contributions

**Tuba Sengul:** conceptualization, methodology, manuscript preparation, manuscript writing, data analysis. **Aleyna Özkan:** data collection, data analysis, literature review, manuscript writing, manuscript editing. **Ayşe Eminoğlu:** data collection, literature review, manuscript writing, interpretation, manuscript review, data analysis, manuscript editing. **Noordeen Shoqirat:** study methodology, data analysis, manuscript editing. **Charleen Singh:** data interpretation, manuscript editing, manuscript revision. **Deema Mahasneh:** study design, manuscript review. **Holly Kirkland‐Kyhn:** manuscript review, academic guidance.

## Ethics Statement

Ethical approval was obtained from Koç University Social Sciences Institutional Review Board (Approval Number: 2022.337.IRB3.153).

## Consent

I have been encouraged to ask questions, and all of my questions have been answered to my satisfaction. By signing this form, I voluntarily agree to participate in this study.

## Conflicts of Interest

The authors declare no conflicts of interest.

## Supporting information


**Appendix S1:** Supporting Information.

**Appendix S2:** Supporting Information.

## Data Availability

The data supporting this study's findings are available upon request from the corresponding author. Due to confidentiality agreements, the data are not publicly accessible.
